# Clinicians’ views of factors of importance for improving the rate of VBAC (vaginal birth after caesarean section): a qualitative study from countries with high VBAC rates

**DOI:** 10.1186/s12884-015-0629-6

**Published:** 2015-08-28

**Authors:** Ingela Lundgren, Evelien van Limbeek, Katri Vehvilainen-Julkunen, Christina Nilsson

**Affiliations:** Institute of Health and Care Sciences, The Sahlgrenska Academy at University of Gothenburg, Box 457, SE-405 30 Gothenburg, Sweden; Department of Midwifery Science, Zuyd University, PO Box 1256, 6201 BG Maastricht, The Netherlands; University of Eastern Finland, Faculty of Health Sciences, Kuopio University Hospital, PO Box 1627, 70211 Kuopio, Finland

## Abstract

**Background:**

The most common reason for caesarean section (CS) is repeat CS following previous CS. Vaginal birth after caesarean section (VBAC) rates vary widely in different healthcare settings and countries. Obtaining deeper knowledge of clinicians’ views on VBAC can help in understanding the factors of importance for increasing VBAC rates. Interview studies with clinicians and women in three countries with high VBAC rates (Finland, Sweden and the Netherlands) and three countries with low VBAC rates (Ireland, Italy and Germany) are part of ‘OptiBIRTH’, an ongoing research project. The study reported here is based on interviews in high VBAC countries. The aim of the study was to investigate the views of clinicians working in countries with high VBAC rates on factors of importance for improving VBAC rates.

**Methods:**

Individual (face-to-face or telephone) interviews and focus group interviews with clinicians (in different maternity care settings) in three countries with high VBAC rates were conducted during 2012–2013. In total, 44 clinicians participated: 26 midwives and 18 obstetricians. Five central questions about VBAC were used and interviews were analysed using content analysis. The analysis was performed in each country in the native language and then translated into English. All data were then analysed together and final categories were validated in each country.

**Results:**

The findings are presented in four main categories with subcategories. First, a common approach is needed, including: feeling confident with VBAC, considering VBAC as the first alternative, communicating well, working in a team, working in accordance with a model and making agreements with the woman. Second, obstetricians need to make the final decision on the mode of delivery while involving women in counselling towards VBAC. Third, a woman who has a previous CS has a similar need for support as other labouring women, but with some extra precautions and additional recommendations for her care. Finally, clinicians should help strengthen women’s trust in VBAC, including building their trust in giving birth vaginally, recognising that giving birth naturally is an empowering experience for women, alleviating fear and offering extra visits to discuss the previous CS, and joining with the woman in a dialogue while leaving the decision about the mode of birth open.

**Conclusions:**

This study shows that, according to midwives and obstetricians from countries with high VBAC rates, the important factors for improving the VBAC rate are related to the structure of the maternity care system in the country, to the cooperation between midwives and obstetricians, and to the care offered during pregnancy and birth. More research on clinicians’ perspectives is needed from countries with low, as well as high, VBAC rates.

## Background

The rates of caesarean section (CS) are increasing globally, and there is widespread concern over the continuing rise because of the higher risks of maternal mortality and morbidity [1, 2]. From a European perspective, Cyprus has the overall highest CS rate, at 52.2 %, followed by Italy, at 38 %. Apart from a slight reduction in Finland and Sweden, CS rates rose throughout Europe between 2004 and 2010. Only the Netherlands, Slovenia, Finland, Sweden, Iceland and Norway had rates below 20 % [1].

The most significant factor contributing to overall increased CS rates is repeat CS following previous CS [3]. However, based on a limited number of randomised controlled trials that compared outcomes for women planning a repeat elective caesarean with women planning a vaginal birth [4], the currently available evidence demonstrates that VBAC is a reasonable and safe option for most women with previous CS [5]. VBAC is associated with lower maternal mortality and less overall morbidity for mothers and babies [5]. Of women who chose a planned VBAC, VBAC success rates ranged from 70 to 87 % (similar to general vaginal birth rates). Despite this fact, in some European countries, many women who had a previous CS will have a routine CS subsequently [1]. VBAC rates are significantly lower in Ireland, Italy and Germany (29–36 %) than those in Finland, Sweden and the Netherlands (45–55 %) [6]. The variation in CS rates across Europe might reflect national, regional and individual clinicians’ attitudes to clinical decision-making, rather than being evidence-based [1, 7].

A systematic review that evaluated non-clinical interventions for increasing the uptake and success of VBAC showed that providing individualised information to women increased the VBAC rate [8]. Other factors that significantly impacted the VBAC rate were interventions targeted towards clinicians rather than women, developing local guidelines, adopting a conservative approach to CS, using opinion leaders and giving feedback to obstetricians on the mode of birth rates [8]. Still, research on clinicians’ views on and barriers to VBAC and their participation in decision-making is lacking. Few qualitative studies of clinicians’ experiences of VBAC have been conducted. Rees et al. [9] interviewed midwives and physicians in England and found that decision aids for women could be a useful adjunct to current antenatal care, with appropriate support from healthcare professionals. A study from the United States [10] showed that the fear of liability, the convenience of having a CS rather than the physician having to remain in the hospital for the whole of the woman’s labour, and the marginalisation of midwives, all led to avoiding VBAC, according to midwives and physicians.

In summary, only a few studies on clinicians’ views of VBAC have been done, none of them from countries with high VBAC rates. Therefore, the aim of this study was to investigate the views of clinicians working in countries with high VBAC rates on factors of importance for improving VBAC rates.

## Methods

This study is a part of the ongoing 4-year OptiBIRTH project, which is funded by the European Union and aims to increase VBAC rates across Europe through enhanced woman-centred maternity care. Interviews from both clinicians’ and women’s perspectives in three countries with high VBAC rates – Finland, Sweden and the Netherlands – and three countries with low VBAC rates – Ireland, Italy and Germany – are part of the project. Antenatal education interventions targeted towards both women and clinicians are currently being tested in a randomised trial in Ireland, Italy and Germany. In this study, findings from interviews with clinicians in the three countries with high VBAC rates are presented.

A qualitative method was used, which is useful when little is known about the phenomenon under study [11]: clinicians’ views of important factors for improving the rates of VBAC. The original study plan was to perform focus group interviews with midwives and obstetricians. The focus group was first mentioned as a market research technique in the 1920s [12], but it has its basis in social science. It can be used to investigate values, attitudes and the complex phenomena that originate from social interaction [13]. Due to practical problems and time constraints in this study, focus groups with midwives and obstetricians could not be performed in all settings; therefore, we used focus groups and individual interviews combined.

When analysing the focus groups and individual interviews, we were influenced by inductive conventional content analysis [14, 15]. Content analysis has a long history in research and can be both quantitative and qualitative [14]. In qualitative content analysis, the aim is to build a model to describe the phenomenon in a conceptual form, derived from the data [15]. The method is useful when no previous studies have looked at the phenomenon or when it is fragmented [15]. Content analysis is a flexible, pragmatic method for developing and extending knowledge of the human experience of health and illness [14].

### Settings

The studies were performed in Finland, Sweden and the Netherlands. Maternity care in Finland and Sweden is free of charge and funded by taxes. In the Netherlands, all costs regarding maternity care are covered by health insurance. However if low-risk women choose a midwife-led hospital birth, they must make a co-payment for the additional costs of the hospital stay. Some insurance plans cover this co-payment. Midwives in all three countries have an independent role and responsibility during normal pregnancy and labour. In Finland and Sweden, almost all births occur in hospitals. Home birth is not included in the healthcare system. When complications occur, an obstetrician takes over the responsibility, but the midwives remain involved in the woman’s care. In the Netherlands, independent practising midwives provide maternity care to healthy women with uncomplicated pregnancies. They refer women to obstetric-led care when there is an increased risk of complications as defined by a national guideline, developed cooperatively by all the professions involved in maternity care. The home birth rate in the Netherlands is about 20 %, but is decreasing. The overall rate of CS for Finland is 16.8 %, the Netherlands 17.0 % and Sweden 17.1 % [1]. The rate for VBAC varies between 45 and 55 % in these countries [6].

### Care for women with a previous CS

In Finland and Sweden, women do not have the right to have a CS performed if there are no medical or obstetric reasons for it. However, individual circumstances – for example, intense fear of childbirth – are sometimes allowed as an indication for CS. In the Netherlands both options are available and counselling includes information on risks associated with VBAC as well as risks associated with elective CS. As an example, according to the law in Sweden, patients have the right to say no to treatments, but no right to receive a treatment based on personal preference only; however, a medical decision should be made in close cooperation with the patient [16]. The Swedish national medical indications on CS at the mother’s request [17] say that CS can be performed when there are no medical or obstetric reasons for it, provided that the individual circumstances are discussed with the obstetrician, and the woman receives both counselling on the CS risk and supportive counselling within a structured care programme.

In Finland, women have regular visits to maternity clinics during pregnancy. Public health nurses or midwives working in the clinics meet the women regularly during pregnancy, as do GPs [18]. The woman visits the hospital clinic to discuss her birth plan during gestational weeks 36–37, and she can talk with the obstetrician then about the mode of birth and other issues.

According to Dutch guidelines, women with a previous CS have regular prenatal visits with their independent midwife in primary care. The women stay under the midwife’s supervision up until week 37 if no complications occur. Then they are referred to the obstetrician for the remainder of the pregnancy, and the birth takes place in hospital under the obstetrician’s supervision. In practice, however, it is not uncommon for women eligible for VBAC to have a prenatal visit with their obstetrician early in the pregnancy as well. In the hospital, obstetrical nurses, midwives and physician assistants also assist with the care.

The Swedish system involves visits on a regular basis to a midwife during pregnancy. If the pregnancy is normal, the midwife has an independent role and consults an obstetrician only if complications occur or if the woman or the midwife has questions regarding medical issues. The guidelines are local; however, women with a previous CS who do not have medical complications of significance for the upcoming birth will be recommended to have a VBAC. Women expressing intense fear of childbirth, or a strong desire for CS, are referred to special ‘fear clinics’ [19]. In Finland as well as Sweden, hospitals have these special clinics for women with fear of childbirth. At the clinics, the woman can discuss issues around the previous and future births with a specially trained midwife. Additionally, the women can be referred to an obstetrician or a psychologist if needed.

### Data collection and participants

Individual face-to-face or telephone interviews and focus group interviews with clinicians were conducted during 2012–2013. In total, 44 clinicians participated: 26 midwives and 18 obstetricians/gynaecologists/physicians. In Finland (FI), data were derived from 12 individual interviews with midwives and eight individual interviews with obstetricians; in the Netherlands (NL), from 11 telephone interviews, with six obstetricians, three midwives working in clinical practice and two midwives working in primary care; and in Sweden (SE), from one focus group with five midwives and three obstetricians, and one focus group with four midwives and one obstetrician. In each country, the interviews were conducted with clinicians in both urban and rural maternity unit settings.

All participants were asked five questions: (1) In your opinion, what are the important factors for VBAC? (2) What are the barriers to VBAC? (3) What is important to you as a professional? (4) What is your view on shared decision-making? and (5) How can women be supported to be confident with VBAC? These questions were agreed by consensus among the participating researchers during a project meeting in September 2012. The same questions were posed in the same order to all the participants, whether the interviews were performed individually or in a focus group.

### Data analysis

The interviews were transcribed verbatim in their native language and analysed using inductive content analysis [15]. The following steps were used during analysis: selecting the units of analysis, making sense of the data as a whole, conducting open coding, using coding sheets, grouping, categorising and abstracting [15]. The units of analysis were parts of the interview texts answering the five questions. Each participating researcher (CN, EvL, KVJ, IL) in the three countries did open coding in their native language, ending up with 5–10 subcategories per question. The subcategories were translated into English by the researchers (IL, EvL, KVJ, CN), checked by a native speaker in English in each country and then sent to the first author (IL). Skype meetings were held to discuss the findings, using English. After those discussions, all data from the three countries were analysed together in order to identify the main categories and subcategories. During this process, the emerging subcategories and main categories were sent back and forth five times between the researchers in each country (EvL, KVJ, CN) and the first author (IL). The first author led the Skype meetings and changed the findings in line with the other researchers’ comments. All four researchers identified the new subcategories and main categories. The researchers then validated the subcategories and main categories by going back to the interview data in each country. All researchers agreed on the final results and validated them by going back to the interview data.

### Ethical considerations and approval

Approval was obtained for the OptiBIRTH project as a whole and from each participating country separately: Medical Ethical Examination Board, Atrium-Orbis-Zuyd, 12 N101 (NL); Regional Ethical Review Board, Gothenburg, 739–12 (SE); and Committee on Research Ethics, University of Eastern Finland, 20/2012 (FI). The participants in each country gave written informed consent.

## Results

The findings are presented in four main categories: a common approach, obstetricians’ final decision on the mode of birth, support during birth, and the strengthening of women’s trust in VBAC. Each category contains a number of subcategories. The findings are supported by quoted comments from the research participants. The coding following each participant comment indicates the participant’s role (midwife or obstetrician), as well as the country the participant is from: Finland (FI), Sweden (SE) or the Netherlands (NL).

### A common approach

The first category that emerged was related to a common approach in caring for the women. This common approach was characterised as follows: VBAC is considered as the first alternative, all clinicians are confident about VBAC, good communication between professionals is required, all clinicians need to work together as a team, and working in accordance with a model and making agreements with the woman is preferable.

### VBAC is considered as the first alternative

According to the professionals, VBAC is considered as the first alternative for women without any medical reasons for CS. All professionals are highly positive about recommending VBAC in their practice.

*I believe it is very clear that the hospitals we work with are also very much advocates of VBAC in the same way we are. So we are in agreement on that.* (Midwife, NL)

*We have here the kind of care culture that we always target towards vaginal birth.* (Obstetrician, FI)

The obstetricians mentioned medical reasons connected to the woman or the baby as reasons to perform a repeat CS. They also indicated several reasons or situations in which they would not perform a VBAC. These reasons varied from an increased risk of uterine rupture (e.g., after two or more caesareans), to signs of imminent uterine rupture during a previous birth, to situations in which the indication for the previous CS is present again. In summary, when obstetricians estimate the chance of a successful VBAC as very low, they plan for a CS. This estimation is based on a combination of the risk of medical complications and the characteristics of the mother.

*If the woman is very overweight, that means a high risk . . . [if she is] massively overweight with previous CS, you need to watch this very carefully. Maybe these are the most complicated patients.* (Obstetrician, FI)

Clinicians in the Netherlands indicated that they believe that the growing number of legal cases in healthcare and the attention given to them in the media lower the threshold for repeat CS. The legal consequences for a clinician are enormous, and the clinicians said that in the future, the fear of a legal case may lead to a decrease in VBAC rates in the Netherlands. They do not want to risk being accused of neglect, although they are convinced VBAC is the best option medically. Clinicians are sued more easily for not doing interventions than for doing unnecessary interventions. The participants from the other two countries did not mention any legal concerns.

*I believe that in the case of trial of labour [after caesarean, or TOLAC], there is a lower threshold for CS because of the tremendous legal implications. The one moment everything is okay, and the next the baby is dead, and then they wonder if this was preventable. Yes, it would be if we gave every woman with a previous CS a repeat CS. I believe this will lead to fewer VBACs in the Netherlands*. (Obstetrician, NL)

### All clinicians are confident about VBAC

The clinicians reported that their confidence about VBAC is influenced by having a common idea and giving guidance in the same way to women. They have to collaborate and share the same view on VBAC to be successful in motivating woman to give birth vaginally. It is important to follow up all women giving birth by VBAC at the clinic and share the results with each other. One advantage is that the care is similar to and consistent with the counselling guidelines that signal that vaginal birth is the primary and safest way to give birth.

*The professionals ‘play’ in the same way: they speak the same language and this talk gradually reaches the woman. This supports successful VBACs, and actually they are mainly very good experiences for the women.* (Obstetrician, FI)

Furthermore, good collaboration is important between prenatal caregivers and caregivers during the birth. It is exemplified by Dutch clinicians saying that primary care midwives and obstetricians from the hospitals should have the same opinion on how to take care of women. Prenatal care for women with previous CS is mostly delivered by primary care midwives and they are more aware of the woman’s needs and wishes. However, they have no role in VBAC, since a birth after CS is under the responsibility of the obstetrician. As a result, good communication and collaboration between caregivers in primary and secondary care is necessary so that when a woman arrives in the hospital, professionals are aware of her needs and wishes.

*If you have good agreements between professionals [from primary and secondary care], then that [a high standard of care] should be attainable*. (Midwife, NL)

Swedish clinicians referred to the necessity of confronting their own attitudes and beliefs about VBAC and the way they as clinicians mediate the information. If midwives and obstetricians do not believe in VBAC, this will be obvious to the women. Clinicians must ask themselves: is there any concrete evidence for being negative about VBAC? Women must not be misled because clinicians have poor knowledge of the evidence for VBAC.

### Good communication between professionals is required

The clinicians expressed that effective collaboration requires good communication between midwives and obstetricians. The Swedish clinicians mentioned that they should adopt a pleasant tone and speak to each other in a friendly and equal way. They said that it is an advantage for a team’s members to know each other well. They also commented that all staff must support and help each other, especially after a difficult childbirth. Moreover, the Dutch clinicians stated that communication needs to be clear; documentation could be improved, according to these clinicians. The clinicians in the Netherlands expressed a need for improved communication. The obstetricians want to be confident that nurses and clinical midwives will inform them about the progress of the birth when they are not in the delivery room. They want to be sure they are called in on time.

*Good collaboration [is needed] between clinical midwives, physician assistants and gynaecologists when speaking of interpretation of the CTG [cardiotocograph]. So if you are not in the room or you cannot see the CTG, you will be summoned [if CTG patterns deviate].* (Obstetrician, NL)

### All clinicians need to work together as a team

Working together as a team was highlighted by the clinicians. Midwives and obstetricians in Sweden agreed that they have to break down obstacles, disregard prestige and work together. The organisation should be flat, without hierarchy. All staff should cooperate and help each other. When new obstetricians are employed, they must adjust to the collaborative atmosphere. Finnish clinicians mentioned the ‘care team’, which includes the midwife and the obstetrician together with the woman, as highly important in supporting their patients.

*In the obstetrical ward, it’s very clear that we are a team. On other wards, it’s a bit more hierarchical, it’s the doctor who decides . . . but here, it’s sort of everybody can work together; it’s a very flat organization. . . . If the midwife in charge is busy and doesn’t have time to be in the office/reception because she must be in the delivery room, then I answer the telephone, meet new patients who arrive or put on a CTG, and you know roughly what needs to be done, and we help each other all together.* (Obstetrician, SE)

### Working in accordance with a model and making agreements with the woman is preferable

Different ways of making agreements with the women were described by the clinicians. Birth plans are used in all three countries. A model can include local instructions for how to consult women with a previous CS, or a structured care programme for professionals during the pregnancy and birth. The Swedish clinicians mentioned in particular two similar models used in two maternity clinics that they referred to as the ‘conference model’ and the ‘face-to-face model’. These models are dependent on close cooperation with special ‘fear teams’: midwives working at the clinic with women experiencing fear of childbirth. Both models include an agreement for the birth or an agreed birth plan between the woman and an obstetrician or a midwife on the ‘fear team’, clearly documented in the woman’s medical records.

*The women dare to give birth when they understand they will not have the same birth again – for example, prolonged labour or something like that.* (Midwife, SE)

The conference model includes a ‘CS conference’, which refers to a meeting at the maternity clinic with only senior obstetricians present. All referrals from antenatal care midwives or doctors regarding elective CS in the region (with or without medical reasons) are evaluated by these obstetricians. Women whose demands for CS are declined will be referred to the ‘fear team’. During individual meetings with the ‘fear team’ midwives, the midwife, together with the woman, set up a birth plan for her. The face-to-face model includes individual meetings at the maternity clinic between the senior obstetrician and the woman (and possibly her partner) concerning the mode of birth. An agreement implies a guarantee of CS if certain conditions during a vaginal birth appear such as lack of pain relief, or prolonged labour. The clinicians believe it is important that doctors who attend such meetings are few in number, are senior and have the same policy on CS. Moreover, these doctors need to follow up the cases after birth and share their knowledge with other clinicians at the clinic.

*We are the only three doctors having this type of face-to-face meeting. We handle the discussions similarly, and it’s an advantage that no matter which doctor the woman sees, she will be treated in the same way. . . . Only the senior obstetricians have these meetings, since discussing such issues requires experience.* (Obstetrician, SE)

The Swedish clinicians mentioned that a typical reason for women demanding a planned CS is a previous emergency CS after prolonged labour with insufficient pain relief. For those women, agreements and birth plans entail a detailed strategy for their labour that outlines such information as their demands for pain relief, the extent of support needed and the indications for performing a CS when progress is slow. It is important that the staff follow the strategy when caring for the woman during labour. The clinicians recommended such agreements because it helps them decide when to change course and perform a CS.

*You have to present another alternative for the women, meaning that they will try [to give birth]. Through this kind of contract, we guarantee them against a repeat of what happened during the last birth. And it’s common that they think there is no other alternative, that the only way to avoid these things happening again is a planned CS. Actually, they are quite surprised and positive, and say, ‘Yes, I think I can do this’.* (Obstetrician, SE)

### Obstetricians’ final decision on the mode of birth

The second category, obstetricians’ final decision on the mode of birth, comprised the following: only professionals can make the final decision, and directive counselling by obstetricians towards VBAC.

### Only professionals can make the final decision

The clinicians were of the opinion that women should participate in decision-making on the mode of birth, but the final decision should be made by a professional with medical knowledge. The clinicians stated that a medical decision has to be made by obstetricians; a decision about performing a CS cannot be entrusted to lay persons. It is a large decision that demands sound knowledge, and commonly, women do not have enough knowledge to make such an assessment.

*That’s about the same thing as if I decide how the plumber should place the pipes in my home . . . or if I should go on a long holiday abroad and beforehand go to the surgeon and say, can I have my appendix removed so I don’t get sick.* (Midwife, SE)

Moreover, if women have the possibility of deciding about CS, the rate of CS should increase; Swedish clinicians thought this situation could lead to unnecessary costs for society. They assumed that if women decide, this could increase their anxiety even more, because the question about CS creates worries in women. Also, women have a right to receive optimal maternity care, and in most cases, that is not a CS. As one clinician said:

*A choice can only be made if the different alternatives are equally valuable.* (Obstetrician, SE)

### Directive counselling by obstetricians towards VBAC

The clinicians described different ‘national’ models for involving women in the decision-making process. The Swedish clinicians suggested the ‘fear clinics’ as a Swedish model of involving women. In Finland, the clinicians emphasised teamwork among the woman, the midwife and the obstetrician towards the same goal. This teamwork is based on collaborative discussion and decision-making among the team members.

*We have a joint discussion at weeks 36–37 with the obstetrician, the midwife and the woman in the clinic. We talk VBAC from our perspectives, and if needed, another visit will be organised. This is a team discussion.* (Obstetrician, FI)

The Dutch clinicians also considered that women’s involvement in the decision-making process is of importance. They had a highly positive attitude towards a shared decision-making process, because women who came to their decision for VBAC as a result of this process were motivated to succeed. Professionals also believe that taking part in decision-making encourages the women to place greater trust in the right choice being made.

*It is always a good thing when people agree with the decision that is made. Then you have patients who start the birth process motivated. I don’t like having people in the delivery room on whom the decision was more or less imposed. You keep on struggling through birth. I prefer to make sure that that [making the decision] is taken care of during pregnancy and that they think we will take the chance [of having a VBAC] together.* (Obstetrician, NL)

### Support during birth

The third category, support during birth, entailed the following: the need for similar treatment support as other labouring women but with some extra precautions, and clinical recommendations for the care of women during VBAC.

## The need for similar treatment and support as other labouring women but with some extra precautions

The Swedish clinicians suggested treating women with a previous CS in the same way as other childbearing women during childbirth, but with some extra precautions. They underlined that midwives and doctors must handle these women carefully, and at the same time see them as normal women in labour and give the usual care as long as the birth is progressing normally. The midwives and doctors should have no doubts about the woman’s capacity to give birth when she is in labour. Doubts can make midwives only half-hearted in their support for women during birth and thereby affect the women negatively. The Swedish clinicians suggested that midwives and obstetricians must follow women with a previous negative experiences of a birth that ended with CS, to achieve own experiences of that these women have the capacity to give birth vaginally without any problems.

*We are strengthened by watching how happy the patients are when it works, and we have the experience of how excellently women give birth, so we are strengthened by this [experience] in our care of all the other [women].* (Midwife, SE)

Also, Dutch and Finnish professionals stressed the need for guidance and support during labour, especially during the active phase of birth. One-to-one guidance during the active phase of birth is of particular importance. The guidance during labour should be focused on motivating women and giving them confidence. Often these women are afraid that the birth process will be very long, because they experienced this the previous time. So professionals have to take care that the women trust them and believe that the obstetrician will intervene on time.

*It is important that women [during labour] are supported in their goal –* ‘*this time, I am going to do it myself*’ *– and that professionals help them with that.* (Midwife, NL)

To give care with extra precautions means to stay alert for signs of complications, but not let complications be the main awareness. Dutch professionals mentioned that at the birth they should be alert for symptoms of imminent uterine rupture, such as sudden abdominal pain. Next to that, the close monitoring of progress is important, because in the case of poor progress, they must be able to intervene quickly.

*Continuous CTG according to protocol is recommended. However, the difficulty with that is the risk for uterine rupture is 1:1000 and so very low. Then I wonder if we should really tie every woman to the bed with fetal monitoring attached and not even allow her to shower for half an hour. I am a little flexible in this.* (Obstetrician, NL)

### Clinical recommendations for the care of women during VBAC

The clinicians gave a number of suggestions for the care of women during VBAC. Being present and giving continuous support, monitoring the birth closely, creating a secure atmosphere for the women, showing that they have control, and giving the women good pain relief and making sure it works are essential aspects of good care during the birth. Waiting for a spontaneous start and allowing the birth to progress in its own time are preferable. The clinicians recommended that professionals adopt a positive manner, motivate and encourage the woman, are careful, listen to their intuition and take potential insights seriously, and be calm and relaxed. If the woman has had a previous emergency CS, the same phase of labour where the CS was performed is critical. Clinicians should be observant and give the woman extra and focused support during this stage. They need to not only look at the CTG to monitor the woman’s contractions, but also check her contractions with their hands. As long as the progression is good, they should not do anything other than the ordinary. They need to inform the woman continuously about the progression of her birth, and explain what is happening and why things are being done. Fluent collaboration between the professionals on the team helps them to do their work more effectively.

### The strengthening of women’s trust in VBAC

The last category, the strengthening of women’s trust in VBAC, includes the following activities: build women’s trust in giving birth vaginally, recognise that giving birth naturally is an empowering experience for women, alleviate fear and offer extra visits about previous CS, and meet the woman in a dialogue and leave the question about the mode of birth open.

### Build women’s trust in giving birth vaginally

One way to strengthen women’s trust in their ability to give birth vaginally is by sending signals that it is possible. The clinicians said that they needed to help women to believe in VBAC. Central is for the women to understand that the second childbirth is different from the first one, and that there are no barriers for her to give birth vaginally in the next pregnancy.

*Support and encouragement should be given to the woman that vaginal birth is going to be successful – this is going to be a good birth experience for the woman. You tell the woman there are no reasons not to end up giving birth vaginally.* (Obstetrician, FI)

According to the clinicians, it is important to give women information about VBAC as soon as possible after the CS, before they are caught in one way of thinking. If possible, clinicians should give the information directly after the woman is back from the surgical ward. The information must be positive, with the clinicians saying that they need to ‘market’ VBAC and prepare the ground for a vaginal birth at the next pregnancy. That this information has been given should be documented in the women’s records. It is an advantage if the woman meets the operating doctor; as an example, a Dutch obstetrician described that he sent a medical report of the birth by CS home with the mother and in this report recommended the mode of delivery for the next pregnancy. In this way, it is clear to the woman, as well as to other professionals, whether or not VBAC will be possible next time.

*I think it’s very important to speak about this immediately after the CS. It’s not certain that she will catch everything I say and can integrate it with the next pregnancy. But somewhere, she’s already heard of this. And I think this is positive when she comes back.* (Midwife, SE)

Information given to women should be evidence based. The information must be given by a person who is confident and in daily contact with the delivery ward. Of central importance is making sure that the woman fully understands and can see the advantages of VBAC.

*Choices must be explained by the obstetrician, and the role is vital in giving updated and reliable information to women.* (Obstetrician, FI)

All discussions with women during pregnancy should be aimed at gaining the women’s trust and confidence. The women must be well informed and have good information on the risks with CS and vaginal birth. The clinicians reported that sufficient preparation is vital in gaining the confidence of women who are willing to have a VBAC. Prenatal meetings are essential in this preparation.

The clinicians described that a very positive experience of a previous CS can be a barrier for VBAC. They were of the opinion that convincing these women to have a VBAC in the current pregnancy can be difficult. The women want the same experience they had the previous time and sometimes demand this from the gynaecologist or obstetrician. If a woman has already decided to have a CS, it can be challenging to convey to her the positive aspects of VBAC.

### Recognise that giving birth naturally is an empowering experience for women

Regarding the ‘psychological’ benefits, clinicians stated that giving birth naturally is an empowering experience for women, and they recognised that the majority of women want to experience vaginal birth at least once in their life. The negative side of this wish is that women who go into labour and eventually need a repeat CS after all could experience feelings of failure because they wanted the vaginal birth.

*It has something to do with a ‘perfect picture’ in a lot of women, but not all of them. A lot of them believe that you have to give birth vaginally at least once in your life.* (Obstetrician, NL)

The clinicians reported that giving birth vaginally can boost women’s self-confidence. The women are proud that they delivered their baby themselves. The professionals also recognised that the impact of this experience is far greater than women can imagine beforehand.

*They really feel like, ‘You see, I can do this!’ and they are very pleased with it. That is also an important reason.* (Obstetrician, NL)

The professionals also mentioned a faster physical recovery after a vaginal birth, and pointed out that this has positive effects on the initiation of breastfeeding, the bonding between mother and child, and women’s ability to participate in day-to-day life much earlier than after CS.

*We talk about safety issues concerning the baby and the mother, as well as alternatives for good pain alleviation; it is easier and quicker to recover from vaginal birth.* (Midwife, FI)

### Alleviate fear and offer extra visits about previous CS

One barrier to VBAC that Swedish and Finnish clinicians described is women’s fear of childbirth because of a negative or even traumatic experience during the previous CS. The clinicians mentioned that in discussions of the fears connected to a previous birth, clinicians should not put any pressure on the woman but should be supportive of her. At the same time, they should go through everything that is troubling the woman. A woman must be given the opportunity to be heard so that she can tell her clinician that she is afraid.

*You need to try to respond to the woman’s fears by listening to what she is saying.* (Midwife, FI)

The Dutch professionals considered it normal for women to be anxious or maybe somewhat frightened of giving birth vaginally. They may even be less self-confident than women were several years ago. The professionals highlighted the need to talk about the fear women have regarding VBAC and to ask about any underlying reasons for the fear. Simply talking with a woman can often reveal significant information. When it is clear what the exact worry is about, the professionals can better prepare the woman in advance by giving tailored advice or information.

*And then I try to differentiate the fear. Is the fear more medical, or is it fear of the pain? And then we look into the possibilities we have to support these women. So if she is extremely afraid of pain, we will inform her about the possibilities for pain relief.* (Obstetrician, NL)

The clinicians stated that midwives and obstetricians must show interest in and care for women’s previous childbirth experiences. A woman needs to understand what happened during the childbirth. It is also important to help her release any lasting emotional blockages regarding the childbirth experience. Swedish clinicians arrange for women to have individual visits about the experience, together with their partner. The Dutch professionals mentioned that the birth process has to be talked through postpartum in order to ease the way for VBAC in future pregnancies. When women understand what happened during labour, they can reason why the same complications do not have to occur in a next pregnancy and realise that there is no reason not to have a vaginal birth.

*I just try to unravel everything that happened [that led to CS] and explain what exactly happened . . . in a way that they understand it. I believe that contributes to them feeling less anxious.* (Midwife, NL)

### Meet the woman in a dialogue and leave the question about the mode of delivery open

Swedish clinicians emphasised that when meeting women who wish for a CS, professionals need to be present and open-minded. The woman has to be met at the point where she is, and the question about the mode of delivery should stay open. The Dutch clinicians also mentioned these factors and recommended that for women who want a repeat CS and are unwilling to consider other options, it is often more effective to say that they can have a planned CS. It makes women stop fighting for what they want, which can open up the discussion later in pregnancy. Some women reconsider their decision and will have a VBAC after all.

*That gives her [the women who does not want VBAC] some peace of mind. We will say: Listen, we understand it was very heavy the previous time; you will have a CS. Maybe you will think differently about it towards your due date, and then you can always try a vaginal birth.* (Midwife, NL)

The Swedish clinicians recommended that women with a psychiatric or psychological history have a CS if they wish. The Dutch clinicians agreed that those women who had either a positive experience of CS or fear of giving birth did not achieve VBAC in most cases. They often had a CS in the end. The clinicians pointed out that women who deny VBAC must not be forced against their will. The clinicians stated that some requests for CS without a medical indication have to be accepted. The Dutch professionals’ opinions were that women who are not motivated to have a VBAC will not succeed and eventually will end up with a CS, possibly an emergency CS.

*If the mother is not confident and doesn’t want to do it, then don’t bother trying, because you won’t succeed.* (Obstetrician, NL)

## Discussion

### Main findings

The main findings from this study are that in order to improve the VABC rate, clinicians need a common approach, and obstetricians should make the final decision on the mode of birth. Furthermore, the clinicians should support women during birth and help strengthen the women’s trust in VBAC.

### Strengths and limitations

The strength of this study is that a deeper understanding of clinicians’ views of VBAC has been reached by using a qualitative approach. For this study, a qualitative approach was suitable because of the complexity of the studied phenomenon. We were unable to find any earlier research on the phenomenon from countries with high VBAC rates.

A further strength of this study, which is also a limitation, is that both focus groups and individual interviews were used. The strength in using two or more methods for data gathering is that they provide greater variation in the data [20]. In addition, the advantage of a focus group is that the participants can discuss and help each other with describing the studied phenomenon from the perspective of the group [13]. In our study, this was from the perspective of two professions, midwives and obstetricians. However, a limitation is that some participants may be invisible as a result of others wielding more influence in the group. In contrast, individual interviews permit all participants to take part in the same way [13]. The limitation of this study is that different methods were used in the participating countries. Focus groups were held in one country only, while individual interviews were conducted in the other two countries.

As for all qualitative studies, the findings must be interpreted in relation to the study’s context [20]. To facilitate transferability to other contexts, the researcher should clearly describe the context, selection and characteristics of the participants, the method or methods of data collection and the process of analysis [15, 20], which we sought to do. However, the context was different in the participating countries, which is a limitation.

### Interpretation

The findings from this study are based on interviews in Finland, Sweden and the Netherlands. It is interesting that all previous studies on VBAC and professionals that we found were from countries with low VBAC rates [8–10, 21]. One of the aims of the OptiBIRTH project, which this study is part of, is to learn from the best. What could professionals from other countries learn from Sweden, Finland and the Netherlands? Important factors in improving the VBAC rate are having a common approach; viewing VBAC as the first alternative; maintaining good communication between professionals; and ensuring all clinicians work together as a team, work in accordance with a model and make agreements with the woman. A common approach is nothing that an obstetrician or midwife could have as an individual, since it must be related to the structure of the maternity care system. It is interesting that Finland and Sweden differ from the Netherlands with regard to the structure of care for women with VBAC. In the Netherlands, VBAC is a responsibility for obstetricians at hospitals, while in Sweden and Finland, VBAC is a responsibility for midwives in hospitals if everything is progressing normally. However, what is similar for these countries is that midwives have an independent responsibility for normal pregnancy and childbirth. The midwives and obstetricians have clear professional responsibilities that may contribute to having a common approach and working as a team. In Sweden, a national health strategy of giving midwives and obstetricians complementary roles in maternity care, as well as equal involvement in setting public health policy, was introduced during the 1800s [22]. The maternal mortality rate in Sweden in the early 20th century was one third that in the United States. The 19th-century decline in maternal mortality largely resulted from improvements in obstetric care, but was also helped along by the national health strategy of giving midwives and doctors complementary roles in maternity care [22]. A similar development in the Netherlands was the introduction of the ‘law of medical practice’ in 1865, where for the first time, responsibilities were formally divided between doctors and midwives for pathological and physiological labour, respectively [23, 24]. Could these clear professional roles have an impact on the current low VABC rates? More research is needed on how the organisation of maternity care, including the professionals’ roles, is related to VBAC and overall CS rates.

The results from this study show that obstetricians should make the final decision on the mode of birth. The women should be involved, but only clinicians can make the final decision, according to the obstetricians and midwives who participated in this study. The fact that the women do not have a right to decide about the mode of birth without individual counselling and a structured care programme [25] is one answer to the high VBAC rates, according to the clinicians. The Swedish obstetricians and midwives who participated in this study thought that the CS rates would increase without these conditions. However, the national recommendations state that women with certain circumstances could have a CS even if there are no medical or obstetric reasons for it [17], which is in line with a relational model of decision-making [26].

A study from New Zealand that entailed interviews with midwives showed that decision-making is influenced by complex human, contextual and political factors [26]. Fear of litigation is one reason for the high VBAC rates [6, 10]. To work in a care system with national guidelines on CS [17, 25] makes it easier for the individual obstetrician and midwife, according to the findings from this study. Only the clinicians from the Netherlands mentioned fear of litigation as a growing problem. Since we did not ask about legal issues, more research is needed on this question. A study of obstetricians’ attitudes to CS in eight European countries (Luxembourg, the Netherlands, Sweden, France, Germany, Italy, Spain and the United Kingdom) found that fear of litigation was less relevant to physicians’ decision-making in Sweden and the Netherlands, a finding consistent with the low medico-legal burden in these countries, according to the authors [27].

The findings from this study show that during the birth, the woman who has a previous CS has a similar need for support as other labouring women, but with a need for some extra precautions. This recommendation is in line with the intrapartum management of TOLAC described by Scott [28], who stated that the care for women undergoing VBAC differs primarily in the need for caution with induction of labour in women with an unfavourable cervix, the avoidance of overstimulation with oxytocin augmentation and surveillance for prompt recognition of the rare case of uterine rupture. The clinical recommendations from our study verify this management, but our data also include suggestions on how to support a woman during a birth – in particular, be present, create a secure atmosphere and give good pain relief. In addition, for the woman who has had a previous emergency CS, the same phase of labour where the CS was performed is critical. Professionals need to be observant and give her extra and focused support during this stage. The importance of support is verified by a meta-analysis showing that continuous support during labour by professionals and non-professional positively influences both the delivery outcome and the woman’s satisfaction with her care [29]. A planned study from Australia will answer the question of whether continuity of midwifery care through pregnancy, labour, birth and the early postnatal period impacts decision-making in the next VBAC [30]. Furthermore, the clinicians pointed out the importance of strengthening the woman’s trust in giving birth vaginally. The clinicians mentioned both the problems that could be connected to VBAC and the strengthening factors. Much of the earlier research on VBAC concerns risk factors [21]. A meta-synthesis of women’s experiences confirmed only focusing risks [21]. Women need evidence-based information on not only the potential risks involved in VBAC, but also the risks of CS, as well as the positive aspects of VBAC [21].

## Conclusions

In order to improve the VBAC rate, according to obstetricians and midwives in countries with high VBAC rates, a common approach includes being confident about VBAC, considering VBAC as the first alternative, working in accordance with a model and making agreements with the woman; in addition, good communication and teamwork are of importance. Women’s trust in VBAC should be strengthened, any fear should be alleviated and extra visits should be offered, since these activities can empower women. The woman should be met in a dialogue, leaving open the question of the mode of delivery. Obstetricians should make the final decision on the mode of birth, while involving women in counselling towards VBAC. During the birth, the woman who has had a previous CS should receive the same treatment and support as other women, but with some extra precautions. The findings in this study indicate that the VBAC rates are related to the structure of the maternity care system in a country, the degree of cooperation between midwives and obstetricians, and the care offered during pregnancy and birth.
